# Intravascular Large B-Cell Lymphoma Diagnosed on Prostate Biopsy: A Case Report

**DOI:** 10.4274/tjh.2013.0090

**Published:** 2014-12-05

**Authors:** Nazan Özsan, Banu Sarsık, Asu Fergün Yılmaz, Adnan Şimşir, Ayhan Dönmez

**Affiliations:** 1 Ege University Faculty of Medicine, Department of Pathology, İzmir, Turkey; 2 Ege University Faculty of Medicine, Department of Hematology, İzmir, Turkey; 3 Ege University Faculty of Medicine, Department of Urology, İzmir, Turkey

**Keywords:** non-Hodgkin lymphoma, Intravascular large B-cell lymphoma, Prostate, Non-germinal center B-cell

## Abstract

Intravascular large B-cell lymphoma (IVLBCL) is a very rare type of non-Hodgkin lymphoma, usually affecting elderly patients and characterized by selective infiltration of neoplastic cells within blood vessels’ lumina. IVLBCL diagnosed with prostatic involvement is extremely rare. We report a patient of 65 years old, having mostly neurological complaints but diagnosed with IVLBCL upon histopathological examination of transurethral prostate resection material, which revealed large neoplastic cell infiltration totally limited within the lumens of small vessels. By immunohistochemistry, neoplastic cell infiltration was positive with MUM1, bcl-6, and bcl-2 and negative with ALK1, CD10, and CD30, with a high Ki-67 proliferation index. CD34 and CD31 staining showed expression in endothelial cells, highlighting the intravascular nature of neoplastic infiltrate. The patient unfortunately refused to receive treatment and died of the disease 8 months after the diagnosis. IVLBCL, though very rare, should be considered in differential diagnosis of all organ biopsies with intravascular infiltration. Further improvements in the understanding of the pathogenesis and biology of this rare type of lymphoma are mandatory.

## Abstract (tr)

İntravasküler büyük B hücreli lenfoma (İVBBHL), non-Hodgkin lenfomaların nadir bir tipidir, genellikle ileri yaşta görülür, ve neoplastik hücrelerin damar lümeni içerisinde seçici infiltrasyonu ile karakterlidir. Prostat tutulumu ile tanı konan İVBBHL olguları ise çok nadirdir. Altmış beş yaşında, daha çok nörolojik şikayetleri olan, ancak transüretral prostat rezeksiyon materyalinin histopatolojik incelemesinde, tamamen küçük damar lümenleri içerisinde sınırlı neoplastik büyük hücre infiltrasyonunun görülmesi ile tanı konan bir olguyu sunuyoruz. Neoplastik hücre infiltrasyonu immunhistokimyasal incelemede MUM1, bcl-6 ve bcl-2 ile pozitif, ALK1, CD10, CD30 ile negatif saptandı, Ki67 proliferasyon indeksi yüksekti. CD34 ve CD31 endotelial hücrelerde pozitif olup, neoplastik infiltrasyonun damar içi yerleşimini belirgin olarak ortaya koydu. Hasta, ne yazık ki tedavi almayı kabul etmedi ve sekiz ay içerisinde hastalık nedeniyle kaybedildi. IVLBCL, az görülmekle birlikte, intravasküler infiltrasyon içeren tüm organ biopsilerinde ayırıcı tanı içerisinde yer almalıdır. Bu nadir lenfoma tipinin patogenez ve biyolojisinin aydınlatılmasına ihtiyaç duyulmaktadır.

## INTRODUCTION

Intravascular large B-cell lymphoma (IVLBCL) is a very rare type of non-Hodgkin lymphoma and is characterized by selective infiltration of neoplastic cells within blood vessels’ lumina. IVLBCL is an aggressive disease, usually affecting elderly patients with a poor prognosis [1].

Prostatic involvement in IVLBCL is extremely rare and has been described in only a few case reports. We hereby report a 65-year-old male patient who presented with neurological symptoms and was diagnosed with IVLBCL based on transurethral prostate resection (p-TUR) material.

## CASE PRESENTATION

A 65-year-old man was admitted to the hospital with complaints of cervical and back pain. His past history revealed no remarkable illnesses, and he had no evidence of human immunodeficiency virus infection or other causes of immunodeficiency.

Cervical spine magnetic resonance (MR) imaging showed signs consistent with degenerative discopathies at multiple levels and an increase in signals at the level of the conus medullaris, consistent with myelitis. Informed consent was obtained.

The patient also had complaints indicating lower urinary tract symptoms, such as difficulty in urinating.

Laboratory findings showed an increase in lactate dehydrogenase (LDH) levels (682 U/L; reference scale: 240-480 U/L) and a mild decrease in platelet count (83,000/µL; reference range: 150,000-450,000/µL). The prostate-specific antigen (PSA) level was in normal limits (PSA: 2.37 ng/mL; reference range: <4 ng/mL; free PSA: 0.374 ng/mL; F.PSA/T.PSA: 0.16; reference: >0.15). Ultrasonography revealed enlargement in prostatic dimensions of 41x40x36 mm in size. p-TUR with spinal anesthesia was performed. After the surgery, the patient developed paraplegia and urinary and fecal incontinence. Lumbar spine MR imaging done after the surgery revealed signs consistent with lumbar spondylolysis, degenerative changes at multiple levels, and an increase of signals in the conus medullaris, which may reveal myelitis. He was then given prednisolone at 1 g/day for 10 days with the diagnosis of myelitis upon neurology consultation.

Histopathological examination of the p-TUR specimen revealed large neoplastic cell infiltration totally limited within the lumens of small vessels in the stroma, while acinar and glandular structures were all benign.

Immunohistochemical analysis was performed on paraffin-embedded tissue sections with an automated stainer (Ventana Benchmark XT, Ventana Medical Systems, Tucson, AZ, USA) according to protocol. A streptavidin-biotin-peroxidase detection system with diaminobenzidine as a chromogen was used to visualize bound secondary antibodies. The intravascular neoplastic cells were positive for CD20 and negative for CD3 and pan-cytokeratin. The following panel of antibody testing was then performed: CD10, bcl-6, bcl-2, IRF4/MUM1, CD30, Ki-67, CD34, ALK, and CD31. The antibodies were scored as positive when >30% of tumor cells showed immunoexpression. Neoplastic cell infiltration was positive with MUM1, bcl-6, and bcl-2 and negative with ALK1, CD10, and CD30. The Ki-67 proliferation index was high, showing expression in about 90% of neoplastic large cells. CD34 and CD31 stainings showed expression in endothelial cells, highlighting the intravascular nature of the neoplastic infiltrate. In situ hybridization analysis was also performed using oligonucleotides complementary to Epstein–Barr early RNA transcripts in tissue sections of paraffin-embedded tissue in the same automated stainer (Ventana Medical Systems), which revealed negative results. The immunohistochemical expressions (IRF4/MUM1 and bcl-6 positivity, with CD10 negativity) revealed a non-germinal center B cell (non-GCB) phenotype when classified according to the Hans criteria [2]. The patient was diagnosed with IVLBCL from the p-TUR material.

The patient was hospitalized in the hematology department with a diagnosis of lymphoma. No enlarged superficial lymph nodes were found on physical examination. Positron emission tomography-computed tomography (PET-CT) showed an increase in fluorodeoxyglucose signals in both kidneys, which was interpreted as consistent with an inflammatory origin. Upper abdominal ultrasonography revealed mild splenomegaly (135 mm). The patient was given prednisolone at 1 g/day for 10 days, when bone marrow biopsy was performed. Bone marrow biopsy revealed no infiltration of lymphoma from either morphology or immunohistochemistry. R-CHOP chemotherapy was planned, but the patient refused to receive therapy and was discharged at his request. He received no therapy for IVLBCL and died from the disease 8 months after the diagnosis.

## DISCUSSION AND REVIEW OF THE LITERATURE

In the current World Health Organization classification of hematopoietic neoplasms, IVLBCL is defined as a rare type of extranodal large B-cell lymphoma characterized by the selective infiltration of neoplastic cells in the lumina of vessels and capillaries, with the exception of large arteries and veins [3]. The disease is widely disseminated in extranodal sites: the bone marrow, central nervous system, skin, lungs, liver, and spleen are the most common sites of involvement [4]. Lymph node infiltration and lymphadenopathy are usually absent [5]. The clinical signs and symptoms are variable, related to the site of involvement. Neoplastic cells are rarely seen in bone marrow and peripheral blood smears and so IVLBCL is very difficult to diagnose; most of the cases reported have been confirmed by autopsy or cutaneous biopsies [6]. IVLBCL diagnosed in prostate specimens is extremely rare in the literature, usually reported as single cases; to the best of our knowledge, this is the 10th such case reported [1,7,8,9,10,11,12]. Furthermore, none of the patients in the study by Murase et al., with a large series of 96 patients, were diagnosed with prostatic involvement, indicating the rarity of infiltration in this site [6].

All reported cases of primary IVLBCL of the prostate are in elderly patients aged above 60 years [10], and the median age defined for all IVLBCL cases is 67 years (range: 13-85 years) [4].

**Figure 1:**
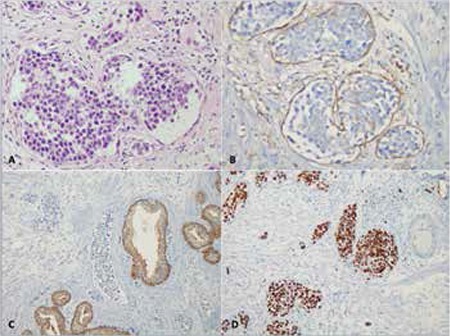
A) Infiltration of neoplastic large cells in the lumen of small vessels in transurethral prostate resection material (hematoxylin and eosin, original magnification: x200) by immunohistochemistry; B) CD31 staining with expression in endothelial cells highlighted the intravascular nature of neoplastic infiltrate (immunoperoxidase, original magnification: x400); C) Neoplastic infiltration is negative with cytokeratine, while benign glandular structures are positive (immunoperoxidase, original magnification: x200); D) Ki67 staining showed a high proliferation index in neoplastic cells (immunoperoxidase, original magnification: x200).

**Figure 2:**
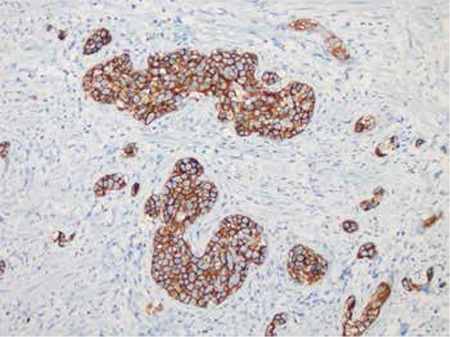
Neoplastic large cells filling the vessels are strongly positive with CD20 (immunoperoxidase, original magnification: x200).

There are no specific laboratory findings indicating IVLBCL, but most patients were reported to display few pathologic findings in their complete blood counts, which should raise suspicion. Ferreri et al., in the study of a series of 38 patients, reported anemia in nearly 65% of patients, increased LDH and β2 microglobulin levels in more than 80% of patients, and an elevated sedimentation component in 14% of patients [1]. Our patient had an increased LDH level and mild thrombocytopenia. He had complaints indicating lower urinary tract symptoms, which led to a urinary tract examination. No increase in PSA levels was found, but ultrasonography revealed enlargement in prostatic dimensions, leading to p-TUR surgery. The diagnosis of IVLBCL was established based on histopathological and immunohistochemical assessment of the biopsy. Neoplastic cell infiltration was totally limited within the lumens of small vessels in the stroma, within benign acinar and glandular structures. Neoplastic cell infiltration was positive with CD20, MUM1, bcl-6, and bcl-2 and negative with ALK1, CD10, CD30, and cytokeratin, with a high Ki-67 proliferation index. CD34 and CD31 staining with expression in endothelial cells highlighted the intravascular nature of the neoplastic infiltrate (Figures 1 and 2). The immunohistochemical profile of infiltration revealed a non-GCB cell phenotype (negative for CD10 and positive for IRF4/MUM1 and bcl-6) when classified according to the Hans criteria. Kanda et al. suggested that most IVLBCL cases might originate from post-germinal center cells, based on the presence of somatic mutation in variable regions of immunoglobulin heavy chain genes [13]. Subsequent studies supported their assertion as most IVLBCL cases have been reported to be of non-GCB cell origin [5,6].

Our patient had no enlarged lymph nodes; bone marrow biopsy showed no infiltration of lymphoma. Upper abdominal ultrasonography revealed mild splenomegaly, but PET-CT showed no findings consistent with extra sites of infiltration; according to these findings, he had stage 1E disease. In a study of 38 intravascular lymphoma patients, Ferreri et al. reported that 40% of the patients diagnosed in vivo had stage 1E disease according to the Ann Arbor staging system, but a disseminated infiltration was shown by autopsy in some stage I disease patients when death occurred a short time after diagnosis, highlighting the limitations of staging procedures in intravascular lymphoma patients [1,14]. The relatively high proportion of false negativity in classical staging procedures can be attributed to the fact that IVLBCL infiltration may usually be seen without tumor masses and apparent signs of involvement or lymphadenopathy [15].

The clinical manifestations of IVLBCL are extremely variable and symptoms are mostly related to the involved organ. Interestingly, clinical manifestations of the disease have been shown to differ between 2 distinct geographical areas. In Asian countries, hemophagocytic syndrome, bone marrow involvement, hepatosplenomegaly, fever, and thrombocytopenia have been documented at high frequencies, while central nervous system and skin involvements are predominantly seen in European countries [1,16]. Despite the defined geographical differences, neurological symptoms at initial diagnosis were reported in high incidences in both the Asian and Western types of the disease at 25% and 36%, respectively [1,17]. Among the few cases reported with prostatic involvement, 2 patients presented with neurological signs indicating spinal cord lesions [8,9]. Our patient had some neurological symptoms interpreted as myelitis according to MR findings, and following p-TUR surgery with spinal anesthesia, paraparesis developed. Whether these neurological symptoms were due to real neurological pathologies or to the invasion of tumor cells into the central nervous system or peripheral nerves remains unclear because an autopsy was not performed.

The disease has an aggressive behavior, usually with a short outcome and fatal course [1]. The use of rituximab-containing chemotherapy regimens for the treatment of IVLBCL has been reported to improve outcomes [17,18].

Ferreri et al. reported overall survival at 3 years as 81% in 33 patients receiving immunochemotherapy [18]. High-dose chemotherapy with the support of autologous stem cell transplantation was shown to have efficacy in several reports [19,20]. Despite all treatment modalities, our patient refused to receive treatment for lymphoma and unfortunately died of the disease 8 months after the diagnosis.

IVLBCL, though very rare, should be considered in differential diagnosis for elderly patients with elevated LDH levels, fever of unknown origin, and unexplained neurological symptoms. As for prostatic involvement, lower urinary tract obstruction symptoms can be seen, but an elevated level of PSA is not a common finding for lymphoma infiltration in the prostate, including rare IVLBCL cases [21]. Biopsy and immunohistochemical assessment are required for the diagnosis. Early diagnosis can result in a better outcome with the recent treatment options. Further improvements in the understanding of the pathogenesis and biology of this rare type of lymphoma are mandatory to achieve better outcomes for IVLBCL patients.

**Conflict of Interest Statement**

The authors of this paper have no conflicts of interest, including specific financial interests, relationships, and/or affiliations relevant to the subject matter or materials included.
